# Chronic exposure to a neonicotinoid pesticide alters the interactions between bumblebees and wild plants

**DOI:** 10.1111/1365-2435.12644

**Published:** 2016-03-14

**Authors:** Dara A. Stanley, Nigel E. Raine

**Affiliations:** ^1^ School of Biological SciencesRoyal Holloway University of LondonEghamTW20 0EXUK; ^2^ Botany and Plant ScienceSchool of Natural Sciences and Ryan InstituteNational University of IrelandGalwayIreland; ^3^ School of Environmental SciencesUniversity of GuelphGuelphONN1G 2W1Canada

**Keywords:** bumble bee *Bombus terrestris*, ecotoxicology, flower visitation, foraging behaviour, insecticide, pollen, pollinator declines

## Abstract

1. Insect pollinators are essential for both the production of a large proportion of world crops and the health of natural ecosystems. As important pollinators, bumblebees must learn to forage on flowers to feed both themselves and provision their colonies.
2. Increased use of pesticides has caused concern over sublethal effects on bees, such as impacts on reproduction or learning ability. However, little is known about how sublethal exposure to field‐realistic levels of pesticide might affect the ability of bees to visit and manipulate flowers.
3. We observed the behaviour of individual bumblebees from colonies chronically exposed to a neonicotinoid pesticide (10 ppb thiamethoxam) or control solutions foraging for the first time on an array of morphologically complex wildflowers (*Lotus corniculatus* and *Trifolium repens*) in an outdoor flight arena.
4. We found that more bees released from pesticide‐treated colonies became foragers, and that they visited more *L. corniculatus* flowers than controls. Interestingly, bees exposed to pesticide collected pollen more often than controls, but control bees learnt to handle flowers efficiently after fewer learning visits than bees exposed to pesticide. There were also different initial floral preferences of our treatment groups; control bees visited a higher proportion of *T. repens* flowers, and bees exposed to pesticide were more likely to choose *L. corniculatus* on their first visit.
5. Our results suggest that the foraging behaviour of bumblebees on real flowers can be altered by sublethal exposure to field‐realistic levels of pesticide. This has implications for the foraging success and persistence of bumblebee colonies, but perhaps more importantly for the interactions between wild plants and flower‐visiting insects and ability of bees to deliver the crucial pollination services to plants necessary for ecosystem functioning.

## Introduction

Bumblebees are important pollinators of both crops and wild plants (Stanley & Stout 2014; Kleijn *et al*. 2015). They forage in the environment to collect nectar and pollen, both to feed themselves but also to provision their colonies and feed their developing brood. An individual worker will continue to forage even when they themselves are satiated, and can forage throughout their entire lifetime (Hagbery & Nieh 2012). In order to forage effectively, bees must be able to learn to locate flowers, assess their profitability and how to manipulate them to extract their rewards. As flowers vary hugely in their salient features for pollinators (including their colour, scent and morphology), there is considerable variation in the range of cues bees must detect and learn. As a result, foraging can be a cognitively challenging task, and foraging on complex flowers is typically more challenging than on simple ones (Laverty 1994). In addition, bees may forage for nectar, pollen or both, and it has been suggested that foraging for pollen can be a more challenging task than foraging for nectar (Raine & Chittka 2007b).

In recent years, declines in bumblebees (Grixti *et al*. 2009; Cameron *et al*. 2011; Dupont, Damgaard & Simonsen 2011) and other pollinators (Biesmeijer *et al*. 2006; Ollerton *et al*. 2014) have led to concern over the use of pesticides in agriculture. Bees can become exposed to pesticides while foraging on treated crops or in treated areas, but typically are exposed at levels that are not lethal. This has resulted in an increasing body of research on the sublethal impacts of pesticides on bees, and a moratorium on the use of three neonicotinoid pesticides as seed treatments for crops attractive to bees in the EU (Regulation (EU) No 485/2013). Neonicotinoids are widely used worldwide and have received much attention in terms of bees due to the risk they pose in comparison to other pesticides (Sanchez‐Bayo & Goka 2014). In addition, they are commonly applied as seed treatments to flowering crops that results in oral exposure of bees foraging on contaminated nectar and pollen. Neonicotinoids are agonists of the nicotinic acetylcholine receptors (nAChRs) and can cause neuronal deactivation in the mushroom bodies of honeybee brains by overexcitation following blocking (Palmer *et al*. 2013; Moffat *et al*. 2015). As the mushroom bodies are linked with both learning and memory (Zars 2000; Menzel 2012), it is unsurprising that impacts of pesticides on learning ability have been established in both honeybees (Decourtye *et al*. 2004a,b, 2005; Williamson, Baker & Wright 2013; Williamson & Wright 2013) and bumblebees (Stanley, Smith & Raine 2015). In addition to direct effects on learning and memory ability, a range of sublethal effects of pesticide exposure on bees have been identified such as impacts on foraging (Gill, Ramos‐Rodriguez & Raine 2012; Schneider *et al*. 2012; Feltham, Park & Goulson 2014; Gill & Raine 2014), navigation (Vandame *et al*. 1995; Fischer *et al*. 2014) and reproduction (Gill, Ramos‐Rodriguez & Raine 2012; Whitehorn *et al*. 2012; Rundlöf *et al*. 2015).

However, there is an increasing call to make research on pesticides and bees more ‘field‐realistic’, using measurements from field trials or experiments as close to field conditions as possible. With this in mind, semi‐field experiments have shown that the impacts of pesticides on learning ability measured in the lab seem to translate into impacts on bee foraging ability in the field. Using RFID technology to measure when bumblebees enter and leave their colony, it has been shown that bees exposed to neonicotinoid pesticides bring back smaller pollen loads or pollen less often, and also behave differently in terms of the amount of time spent foraging (Gill, Ramos‐Rodriguez & Raine 2012; Feltham, Park & Goulson 2014; Gill & Raine 2014). Although this evidence suggests that pesticide exposure can alter the ability of bees to forage and manipulate flowers, direct observations of flower‐visiting behaviour are lacking. Whilst it has been shown that pesticide exposure can alter flower visitation patterns to apples, a commercial crop with simple floral morphology (Stanley *et al*. 2015), it is not known whether this may also be the case for wild plants with more complex floral morphology.

**Figure 1:**
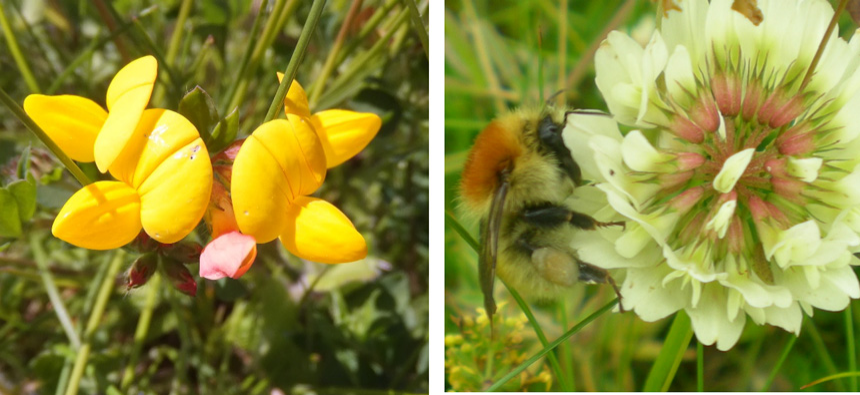
Complex morphology of *Lotus corniculatus* (bird's foot trefoil; left) and *Trifolium repens* (white clover; right; being visited by the large carder bee, *Bombus muscorum*). Photos by DAS.

Here, we investigated whether pesticide exposure can cause changes in the ability of bumblebees to learn how to manipulate and forage from morphologically complex flowers (Laverty 1994). To do this, we allowed naive individual bumblebees (from colonies pre‐exposed chronically to either pesticide or control solutions) access from their colony to a flight arena provisioned with complex flowers of *Lotus corniculatus* L. (bird's foot trefoil) and *Trifolium repens* L. (white clover; Fig. 1), both species commonly encountered by bumblebees in agricultural areas (Carvell *et al*. 2006). We then recorded their flower visitation and foraging behaviour.

## Materials and methods

*Lotus corniculatus* and *T. repens* were obtained as plant plugs (from British Wild Flower Plants, Fig. 1), and potted into larger pots in March 2014. Ten colonies of *Bombus terrestris audax* were obtained from Biobest (Westerlo, Belgium) in the middle of June, with a queen and an average of 109 workers (range 87–127). On arrival, colonies were transferred to bipartite wooden nest boxes (28 × 16 × 11 cm); the brood in the rear chamber, and the front chamber was used for feeding. The 10 colonies were ranked in terms of number of workers and split into five pairs (blocks), and treatment was randomly assigned within block.

We chose to investigate impacts of the neonicotinoid pesticide thiamethoxam, which was the most widely applied neonicotinoid pesticide on oilseed rape crops in the UK in 2012 (Garthwaite *et al*. 2012), on foraging behaviour. Most studies on the potential effects of neonicotinoids on bees have investigated impacts of another compound, imidacloprid (Decourtye *et al*. 2004a; Laycock *et al*. 2012; Bryden *et al*. 2013; Gill & Raine 2014). However, it has been suggested that impacts of neonicotinoid pesticides may not be the same (Goulson 2013), and that in particular thiamethoxam may be less toxic to bees than imidacloprid (Iwasa *et al*. 2004; Mommaerts *et al*. 2010; Blacquière *et al*. 2012; Laycock *et al*. 2014). A solution of 10 parts per billion (ppb) thiamethoxam was prepared by dissolving 10 mg thiamethoxam (Sigma Aldrich) in 100 mL acetone, then 10 μL of this stock solution was added to 1 L of 40% sucrose solution (these calculations are carried out on a v/v basis; on a w/w basis this would give a solution of 8·5 ppb thiamethoxam). The same process was repeated using 10 μL acetone only to produce an equivalent control solution. Solutions were stored in a dark refrigerator for up to 7 days, after which a new batch was prepared to ensure consistent pesticide concentrations. We chose to use 10 ppb thiamethoxam as this falls within the range of neonicotinoid concentrations measured in plant residues under field conditions (Castle *et al*. 2005; Dively & Kamel 2012; Stoner & Eitzer 2012; Godfray *et al*. 2014, 2015; Stewart *et al*. 2014; Botías *et al*. 2015; Rundlöf *et al*. 2015) and is comparable to previous work (Gill, Ramos‐Rodriguez & Raine 2012; Laycock *et al*. 2012, 2014; Stanley *et al*. 2015). Every 2 days, a new colony pair began treatment with either 10 ppb thiamethoxam in sugar water or control sugar water (prepared as explained above), to minimize potential for intercolony variation in duration of the pesticide exposure. Colonies were fed both their treatment sucrose solution and untreated commercial honeybee collected pollen (that had previously been frozen) every 2 days. The majority of sugar water was consumed and bees had no alternative food source for a 9–10 day period; therefore any workers tested would have fed on their treatment solution.

Colonies were tested after 9 or 10 days of pesticide exposure. This length of time was chosen to mimic a situation where bees fed on oilseed rape and/or contaminated wild plants exclusively during peak flowering period of the crop. Prior to testing, each colony was allowed access to a gravity feeder (containing their treatment solution) in a flight arena (60 × 35 × 100 cm) to encourage foraging behaviour for 48 h. On the day of testing, each block was connected to a large flight arena (78 × 52 × 100 cm) in a bright but shaded outdoor location. Flight arenas were provisioned with two flowering *L. corniculatus* plants (with an average of 131 florets across both plants per day) and one flowering *T. repens* (average 11 flowering inflorescences per day; the term flower will subsequently be used to signify *L. corniculatus* florets and *T. repens* inflorescences). These species were chosen as they are known to be important forage plants for bumblebees (Carvell *et al*. 2006), and their flowers have complex morphology (Fig. 1) making them relatively difficult for bumblebees to learn how to handle to extract nectar and pollen. The number of flowers provided by each species was standardized across pairs so each colony in the pair (block) was exposed to the same floral density on each day.

Bees were allowed to enter the flight arena one at a time and the foraging behaviour of each bee was recorded individually by an observer (DAS or DW) using Etholog software (Ottoni 2011). This allowed us to record the number of flowers of each species visited, the time taken to handle each flower, whether individuals collected pollen (or not) and the size of pollen loads (classified as either ‘small’, ‘medium’ or ‘large’). We also judged when a bee had properly ‘learnt’ to manipulate a flower (i.e. when a bee landed on a flower and immediately collected nectar and/or pollen, without exploring the flower first; this was not recorded for all bees as in some cases the transition was not obvious). Each bee was observed for 30 min or until it tried to return to the colony, whichever was sooner. At the end of each observation period, tested individuals were placed into a plastic vial and frozen for subsequent measurement of body size. Individuals that did not visit any flowers within 20 min were assumed not to be foragers and removed, and the next bee released. A 10‐min break was taken between testing foragers to allow dissipation of any scent marks and replenishment of nectar in the flowers (Stout, Goulson & Allen 1998). Each colony was observed for 2 days, and plants were changed each day. The treatment of the colony observed was unknown by one of the observers, although the other was aware of treatment as they were also responsible for managing and feeding colonies in the lab. Observations were carried out from 23 June until 3 July, between 1030 and 1600. After the experimental period, we measured the thorax width (as a proxy for body size) of all tested bees using digital callipers.

A number of measures of behaviour were extracted from the Etholog data sets: (i) the length of time spent foraging (the time elapsed between the first and last flower visit); (ii) the average length of time between flower visits; (iii) the average visit length to each flower species; (iv) the amount of time it took each bee to learn proper foraging behaviour (as defined above; when a bee immediately went for nectar and/or pollen rather than exploring the flower first); (v) the total number of flowers of each species visited separately; (vi) the number of switches between flower species; (vii) the number of flowers visited before proper foraging behaviour was learnt; (viii) whether bees visited *L. corniculatus* or *T. repens* first; (ix) the proportion of visits to *T. repens* and (x) the proportion of bees that foraged for pollen. We investigated treatment (pesticide‐exposed vs. control) differences in these behavioural measures of foragers using linear mixed effects models in R (R Development Core Team 2011). We used the lme function from the nlme package for models in which time was the response variable (Pinheiro *et al*. 2012), the glmer function from the lme4 package for any response variables that were counts or proportions (with poisson or binomial distributions specified: (Bates *et al*. 2014)), and the glmmPQL function from the MASS package for any models where data were overdispersed (Venables & Ripley 2002). To account for any differences in behaviour caused by weather conditions or other interdiurnal differences, date of testing (nested within block) was included as a random effect. The body size of bees was included as a covariate, and models were simplified by removing this term if it was not significant. Models were validated by inspecting qq‐plots and histograms of residuals, and plotting standardized residuals vs. fitted values, and data were transformed (log X+1) if necessary to improve model fit.

## Results

In total, 160 bees were observed leaving their colonies to enter the flight arena (average 15 per colony from pesticide colonies, and 17 per colony from control colonies; no difference in numbers of bees released between treatments, quasipoisson glm: F_1,8_ = 4·14, *P* = 0·08) of which 74 bees (46%) were classed as ‘foragers’ (we classified a bee as a forager if it landed on five or more flowers during its time in the arena). A significantly greater number of bees active in the flight arena were foragers in pesticide‐treated colonies (63% of bees per colony for pesticide‐treated, 33% per colony for control colonies; glmer: χ^2^ = 4·9044, *P* = 0·03), but worker body size did not differ between treatments (GLM: F_1,68_ = 0·0277, *P* = 0·87).

**Figure 2:**
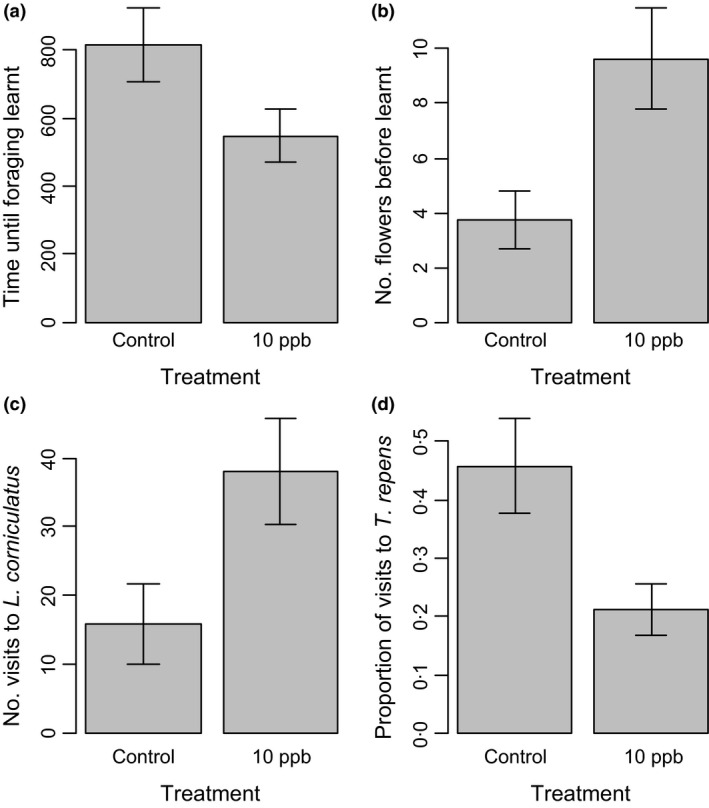
The differences in (a); top left time until foraging behaviour was learnt, (b); top right number of flowers visited before the individual bee learnt to properly manipulate the flower, (c); bottom left number of visits to *Lotus corniculatus* and (d); bottom right proportion of visits to *Trifolium repens*, for individual bees exposed to control (untreated) or 10 ppb thiamethoxam treatments. Columns show means (±SEM) across all foragers observed (47 individuals from 10 ppb colonies, 27 individuals from control colonies for number and proportion of visits to species, and 22 individuals from 10 ppb colonies and 11 from control colonies for time and number of flowers until foraging behaviour learnt). There was a significant difference (*P* < 0·05) between treatments for all variables displayed (Table 1)

There was no difference between treatments in terms of how long bees spent foraging (Table 1), how long they took to handle either species of flower, or the amount of time spent between flower visits (Table 1). Interestingly, although bees exposed to pesticide learnt to manipulate flowers earlier on in their time in the foraging arena, control bees learnt how to manipulate flowers after fewer learning visits than bees exposed to pesticide (Table 1, Fig. 2). Most bees foraged only for nectar, with only 23 of 73 individuals collecting pollen. We found that significantly more bees exposed to pesticide foraged for pollen than control bees (Table 1). All seven of the bees classified as carrying ‘medium’ sized pollen loads were from pesticide‐exposed colonies, while the 15 bees with ‘small’ loads came from both treatment groups (11 pesticide and four control bees).

**Table 1:** Summary of variables measured in observations of individuals from pesticide colonies and control colonies. *n* = 47 foragers from five pesticide colonies, and 27 foragers from five control colonies (except for ‘time taken to for foraging behaviour to be learnt’ and ‘number of flowers visited before foraging behaviour was learnt’ where *n* = 22 foragers from four pesticide colonies, and 11 foragers from four control colonies)

Variable	Mean ± SEM		Model	
	Control	10 ppb	Treatment	Width
Length of time spent foraging	850·79 ± 81·07	940·09 ± 89·38	χ^2^ = 0·76, *P* = 0·38	χ^2^ = 2·68, *P* = 0·10
Length of time spent between flower visits^b^	35·51 ± 5·24	31·55 ± 5·26	χ^2^ = 1·31, *P* = 0·25	
Mean visit length to *L. corniculatus*	7·52 ± 1·06	6·9 ± 0·66	χ^2^ = 0·1, *P* = 0·76	
Mean visit length to *T. repens*	27·77 ± 4·44	23·82 ± 3·56	χ^2^ = 0·18, *P* = 0·67	
Time until foraging behaviour was learnt^b^	815·21 ± 107·91	549·35 ± 78·77	**χ** ^**2**^ = 4·32, *P* = 0·04^a^	
No. of visits to *L. corniculatus* b	15·81 ± 5·84	38·02 ± 7·62	**χ** ^**2**^ = 9·8, *P* = 0·002^a^	
No. of visits to *T. repens* b	7·52 ± 1·71	5·87 ± 1·64	χ^2^ = 0·07, *P* = 0·79	χ^2^ = 0·47, *P* = 0·49
No. of switches between flower varieties^b^	1·1 ± 0·28	1·7 ± 0·46	χ^2^ = 0·85, *P* = 0·36	χ^2^ = 2·90, *P* = 0·09
No. flowers visited before foraging behaviour learnt	3·7 ± 1·06	9·6 ± 1·8	**χ** ^**2**^ = 7·3, *P* = 0·007^a^	
Proportion of bees that visited *L. corniculatus* first	0·52	0·81	**χ** ^**2**^ = 6·54, *P* = 0·01^a^	
Proportion of visits to *T. repens*	0·46	0·21	**χ** ^**2**^ = 6·24, *P* = 0·01^a^	
Proportion of bees that foraged for pollen	0·15	0·39	**χ** ^**2**^ = 4·53, *P* = 0·03^a^	

All times given are in seconds. Values given are means (± S.E.M.) across all individuals released.

a indicates significant differences (*P* < 0.05).

b Indicates data were transformed for analysis.

Bees exposed to pesticide visited more *L. corniculatus* flowers than control bees (Table 1, Fig. 2, Table S1, Supporting information), although there was no difference in the number of *T. repens* flowers visited between treatment groups; however, this meant that a higher proportion of visits by control bees were to *T. repens*. Interestingly, there was a trend towards a preference of pesticide‐exposed bees to visit a *L. corniculatus* flower first rather than a *T. repens* (13 of 27 control bees (48%) first landed on *T. repens*, whereas only 9 of 47 pesticide‐exposed bees (19%) chose *T. repens* first; Table S1), although this was not significant. There was no difference in the frequency with which bees from each treatment switched between flower species (Table 1).

## Discussion

We found that chronic exposure to field‐realistic levels of thiamethoxam altered the interactions between bumblebees and morphologically complex wildflowers. First, a higher proportion of bees that were released from pesticide‐treated colonies became foragers in comparison to control colonies. Of these foragers, bees exposed to pesticide visited more *L. corniculatus* flowers, showed a trend towards a preference for this species on their first flower visit and collected more pollen. However, although bees exposed to pesticide learnt to manipulate flowers sooner, control bees learnt to manipulate flowers after fewer flower visits than pesticide‐exposed bees, and also visited a higher proportion of *T. repens* flowers.

Interestingly, we see increased activity in bees exposed to pesticide in terms of the numbers of *L. corniculatus* flowers visited. This is similar to work showing bees visit a higher number of apple flowers when exposed to field‐realistic thiamethoxam levels (Stanley *et al*. 2015), a result that may be indicative of hormesis; a stimulation of biological processes at low doses (Cutler & Rix 2015). Other putative hormetic effects have been found following exposure to other neonicotinoids: imidacloprid, in combination with the acaricide coumaphos, can cause modest improvement in honeybee learning and memory (Williamson, Baker & Wright 2013) and exposure to low‐levels of clothianadin can lead to improved orientation behaviour in moths (Rabhi *et al*. 2014). However, although individual bees visited more flowers in the Stanley *et al*. (2015) study, the pollination services provided were not affected suggesting that this increased activity did not deliver improved pollination quality.

Previous studies of colonies foraging freely outside in the field have found that bees exposed to imdacloprid bring back pollen less often (Feltham, Park & Goulson 2014) and/or bring back smaller pollen loads (Gill, Ramos‐Rodriguez & Raine 2012). Here, we find bees exposed to similar levels of thiamethoxam actually bring back pollen more often than controls. This may be related to the decreased amount of time spent learning how to manipulate flowers, allowing pesticide‐exposed bees more time to collect pollen (see additional discussion of speed‐accuracy trade‐offs below). However, this pattern may change over time, as bees exposed to imidacloprid have been shown not to improve their foraging ability over time – unlike, unexposed, control bees (Gill & Raine 2014). In addition, our data were collected in an outdoor flight arena in which bees had to fly less than 50 cm to access their first flower, representing a relatively simple environment with little need to navigate, locate forage resources or avoid predators. Previous studies were carried out in a natural, outdoor setting (Gill, Ramos‐Rodriguez & Raine 2012; Feltham, Park & Goulson 2014), with bees facing a much more challenging environment in terms of navigation and location of floral resources. This could indicate that impairments in foraging ability following pesticide exposure may not be due to patterns of flower visitation, but the ability of bees to deal with variation in weather conditions, landscape‐scale navigational complexity or indeed responses to additional stressors in the environment.

Although pesticide‐exposed bees collected pollen more often and visited more flowers overall, we found that control bees visited fewer flowers before manipulation behaviour was learnt. As bumblebees display trade‐offs between the speed and accuracy with which they make foraging decisions (Chittka *et al*. 2003; Ings & Chittka 2008; Chittka, Skorupski & Raine 2009), and exposure to pesticides can affect learning and memory performance in bumblebees (Stanley, Smith & Raine 2015), it is also possible such exposure could affect speed‐accuracy trade‐offs. Bees exposed to pesticide initially forage faster and collect more pollen as control bees might be investing more time and/or energy in learning. It can take up to 30 foraging trips for an individual bee to reach maximum foraging efficiency (Peat & Goulson 2005), and the average handling times for *L. corniculatus* measured here on a first foraging bout are higher than those measured for experienced bees in the field (Stout & Goulson 2002). Therefore as we only observed the first foraging trip, control bees had not yet fully learnt how to forage to the best of their ability, and so may not yet have been ‘accurate’ foragers. This view is supported by previous work showing that bees exposed to (imidacloprid) pesticide do not improve their pollen collection performance over time but un‐exposed bees do (Gill & Raine 2014).

We found a difference in floral preferences between our treatment groups; pesticide‐exposed bees exposed visited more *L. corniculatus* flowers and were more likely to visit this species first, but control bees visited a higher proportion of *T. repens* flowers. Previous work has also found differences in the colour of pollen loads collected by imidacloprid‐exposed bees compared with untreated controls (Gill & Raine 2014), suggesting impacts of pesticides on floral preference. A mechanism for this could be detrimental impacts of pesticide on cognition (Stanley, Smith & Raine 2015), particularly the ability to learn to manipulate a greater number of flower types ‐ a task known to be more cognitively challenging (Gegear & Laverty 1995, 1998). *Lotus corniculatus* and *T. repens* differ in colour, morphology (Fig. 1) and quantity of rewards (with *L. corniculatus* producing more nectar than *T. repens*; Raine & Chittka 2007a), all of which may affect how bees learn to manipulate them. However, *T. repens* is a more nutritious forage source than *L. corniculatus* with twice the total sugar content and higher concentrations of amino acids (E. Power, personal communication). The nutritive quality of floral resources can influence bee foraging behaviour (Somme *et al*. 2015); therefore another mechanism could be that pesticide may influence a bee's ability to choose forage resources based on nutritive content (although bees cannot taste neonicotinoids Kessler *et al*. 2015). These changes in floral preference may be the cause of differences seen in other measures in our study, such as length of time spent foraging. However, to fully disentangle these effects of species choice and arrangement, bees would have to be presented with both species singly and as mixtures which would be a useful follow‐on experiment from this study.

Although, to our knowledge, this study is the first to investigate impacts of pesticides on foraging behaviour of bees on real wildflowers, some previous studies have investigated similar impacts using artificial food sources in the laboratory. Using RFID technology in a flight arena, honeybees exposed to imidacloprid and clothianadin showed a reduction of foraging activity and longer foraging bouts when exposed to high pesticide concentrations, although with no impact seen at field‐realistic levels. (Schneider *et al*. 2012). Morandin & Winston (2003) found that bumblebees (*Bombus impatiens*) exposed to 7 ppb imidacloprid in pollen had a similar foraging rate to untreated controls, but that bees exposed to higher levels (30 ppb) had a significantly lower foraging rate. Using comparable doses of another neonicotinoid, clothianadin, Franklin, Winston & Morandin (2004) found no difference in times taken by pesticide‐treated and control bees to access rewards from artificial flowers in a foraging arena after 48 days of exposure, although there was a trend towards lower mean access times for bees exposed to 6 ppb and 36 ppb. However, it is likely that visitation to real flowers with complex morphology represents a significantly more challenging task to bees than foraging on simple artificial flowers, and our work suggests that under these conditions impacts on foraging behaviour may be more apparent.

Changes in foraging behaviour resulting from pesticide exposure are interesting from the ‘bee’ perspective as it introduces the potential to alter colony provisioning that places additional stress on the colony with implications for colony survival (Bryden *et al*. 2013). However, bees provide essential pollination services to crops and wild plants (Klein *et al*. 2007; Ollerton, Winfree & Tarrant 2011), and as such changes in foraging behaviour may have knock‐on impacts for the pollination services they deliver. Although pesticide exposure has been shown to decrease pollination services delivered to apple crops (Stanley *et al*. 2015), the extent to which this might also be true for wild plants is unclear. An increase in numbers of foragers, thereby making more flower visits and collecting more pollen (and hence transporting more pollen between individual plants), may have positive implications for the delivery of pollen to flowers and therefore seed set. Alternatively, if bees exposed to pesticide take longer to learn to manipulate flowers and show different floral preferences, or scent mark flowers without proper visitation thereby discouraging other bees from visiting them (Stout, Goulson & Allen 1998; Stout & Goulson 2002), this could have negative impacts on pollination service delivery.

The majority of research on the impacts of neonicotinoids on bees to date has focussed on imidacloprid, using honeybees as a model system (Godfray *et al*. 2014, 2015; Lundin *et al*. 2015). Here, we find that field‐realistic levels of thiamethoxam can alter foraging behaviour of bumblebees in a relatively simple environment. At similar exposure levels of thiamethoxam, effects on bumblebee reproduction seem to be variable; at 10 ppb nest building was delayed and no larvae were produced (Elston, Thompson & Walters 2013), no detectable effect on reproduction or survival of queenless microcolonies was detected at 11 ppb (Laycock *et al*. 2014) or on male production at 10 ppb (Mommaerts *et al*. 2010). However, following chronic exposure to 10 ppb thiamethoxam bumblebees learn an olfactory conditioning task more slowly than controls and their short term memory can be affected (Stanley, Smith & Raine 2015). This suggests that it could be useful to incorporate other behaviours, such as learning ability and foraging, into pesticide risk assessments that currently use only mortality or reproduction; impacts may be seen on foraging when no impacts on reproduction are detectable (Mommaerts *et al*. 2010).

There are a number of environmental stressors that can cause changes in bee foraging behaviour (e.g. parasites; Schmid‐Hempel & Stauffer 1998; Gegear, Otterstatter & Thomson 2005; Otterstatter *et al*. 2005; invasive species; Dohzono *et al*. 2008; predators: Jones & Dornhaus 2011). Our work shows that exposure to field‐realistic levels of pesticide stress can also alter foraging behaviour of bumblebees on real wildflowers with complex morphology even in a relatively unchallenging scenario. This suggests that under more challenging conditions in a wild, fully‐outdoor setting, impacts may be augmented. As we only looked at the first foraging bout of individuals, it is likely that impacts may also change over the foraging life of the individual. Our work highlights the need to include taxa other than honeybees in risk assessments for pesticide use, and that bumblebees can also be a useful study taxon. It also confirms that changes in foraging behaviour on wildflowers represent another sublethal impact of pesticide use, which may have implications for the delivery of pollination services to wild plants.

## Author contributions

DAS and NER conceived the project, designed the experiment and wrote the manuscript. DAS carried out the experiment and statistical analyses.

## Data accessibility

Data are given in the Supporting Information.

## Supporting information


**Lay Summary**

**Table S1.** Sequences and flower handling times (in seconds) of the first 30 floral choices for all foragers exposed to control (a) or pesticide (10 ppb thiamethoxam) (b) treatments; n  =  the total number of flowers visited in the foraging bout. Light grey represents visits to *Lotus corniculatus*, and dark grey represents visits to *Trifolium repens*.
