# Supplementary material for: Chronic exposure to a neonicotinoid pesticide alters the interactions between bumblebees and wild plants
Source: Funct Ecol. 2016 Mar 14;30(7):1132–9. doi: 10.1111/1365-2435.12644 (PMC4950133; doi:10.1111/1365-2435.12644)
Supplement: Supplementary file 1 — Lay Summary [file FEC-30-1132-s001.pdf]

Functional Ecology
British Ecological Society

Chronic exposure to a neonicotinoid pesticide alters the interactions between bumblebees and wild plants

Dara A. Stanley & Nigel E. Raine

Bees are crucially important for humans and for nature, as they pollinate our crops and also the majority of flowering wild plants. However, bee declines have been recorded worldwide, with concerns for the continued provision of these pollination services. Neonicotinoid pesticides, one of the most widely used pesticide groups in the world, are one factor implicated in the decline of bees. These pesticides affect regions of the bee brain associated with learning and memory, and therefore have the potential to alter how bees visit flowers.

Our experiment tested whether exposure to a neonicotinoid pesticide, thiamethoxam, affected how bees visit flowers of two wild plant species, birds foot trefoil and white clover. We exposed bumblebee colonies to a field-realistic level of pesticide (10ppb thiamethoxam) in the lab for 10 days, and then gave individual bees access on their first foraging trip to a flight arena containing the two plant species where we analysed their behaviour.

We found that bees exposed to pesticide visited more birds foot trefoil flowers than controls, and they collected more pollen. However, they were slower to learn how to properly manipulate flowers. This suggests that control bees may have been investing more time in properly learning how to work flowers,

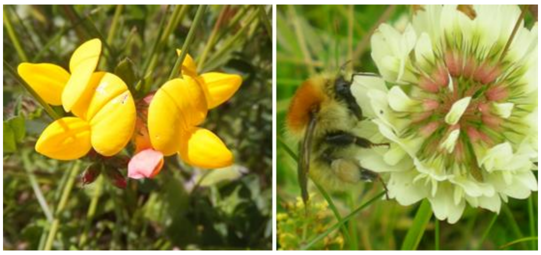

Wildflowers. Image provided by authors.

which in the longer term may be a more viable strategy.

We also found that bees exposed to pesticide seemed to prefer different flowers to controls; pesticide exposed bees visited less white clover, and were more likely to visit birds foot trefoil first. This suggests that pesticide may change the floral preference of bees.

Our work adds to the growing body of evidence examining the impacts of pesticides on bees, but importantly shows that pesticides have the potential to alter the interactions between bees and wildflowers. This is important, as it may result in changes in the pollination services delivered, with consequences for plant reproduction and the functioning of natural ecosystems.
