# Supplementary material for: Chronic exposure to a neonicotinoid pesticide alters the interactions between bumblebees and wild plants
Source: Funct Ecol. 2016 Mar 14;30(7):1132–9. doi: 10.1111/1365-2435.12644 (PMC4950133; doi:10.1111/1365-2435.12644)
Supplement: Supplementary file 2 — Table S1. Sequences and flower handling times (in seconds) of the first 30 floral choices for all foragers exposed to control (a) or pesticide (10 ppb thiamethoxam) (b) treatments; n = the total number of flowers visited in the foraging bout. Light grey represents visits to Lotus corniculatus, and dark grey represents visits to Trifolium repens. [file FEC-30-1132-s002.docx]

**Chronic exposure to a neonicotinoid pesticide alters the interactions between bumblebees and wild plants**

**Supporting Information**

**Dara A. Stanley^1,2*^ & Nigel E. Raine^1,3^**

**^1^** School of Biological Sciences, Royal Holloway University of London, Egham, TW20 0EX, UK; **^2^** Botany and Plant Science, School of Natural Sciences and Ryan Institute, National University of Ireland, Galway, Ireland; ^3^ School of Environmental Sciences, University of Guelph, Guelph, Ontario, N1G 2W1, Canada

*corresponding author: darastanley@gmail.com

**Table S1.** Sequences and flower handling times (in seconds) of the first 30 floral choices for all foragers exposed to control (a) or pesticide (10ppb thiamethoxam) (b) treatments; n = the total number of flowers visited in the foraging bout. Light grey represents visits to *Lotus corniculatus*, and dark grey represents visits to *Trifolium repens*.
